# A242 DELAYED STEROID TAPER MAY REDUCE RISK OF RELAPSE IN PATIENTS WITH IMMUNE CHECKPOINT INHIBITOR ASSOCIATED HEPATOTOXICIT

**DOI:** 10.1093/jcag/gwac036.242

**Published:** 2023-03-07

**Authors:** F Almousawi, M Butler, P Bedard, A Spreafico, L Siu, M Cunningham

**Affiliations:** 1 University of Toronto; 2 Princess Margaret Cancer Centre, UHN, Toronto , Canada

## Abstract

**Background:**

Immune checkpoint inhibitors (ICI) have become the cornerstone of treatment of certain malignancies. However, they can result in systemic toxicities including hepatitis. Societal guidelines recommend initial management with high dose steroids, then a slow taper as hepatitis resolves. However, there is significant variation in steroid response with some patients experiencing a relapse of hepatitis as steroid doses are tapered (“steroid relapse”).

**Purpose:**

Identify clinical features that predict relapse, and to explore variations in steroid management, in patients with ICI hepatotoxicity.

**Method:**

Patients receiving ICI in early phase clinical trials at Princess Margaret Cancer Centre, or treated at the Toronto Centre for Liver Disease for ICI hepatotoxicity, were included. Patients with CTCAE Grade (G)3 ICI hepatotoxicity (ALT >5 x ULN) were identified and clinical records reviewed for management and outcomes. Patients with an alternate cause for ALT elevation; who did not receive corticosteroids; or with HCC or viral hepatitis, were excluded.

**Result(s):**

Between August 2012 and December 2021, 36 patients with G3 ICI hepatotoxicity were identified. Most (23; 64%) had metastatic melanoma. Thirteen received anti-CTLA-4/PD-1; 18 anti-PD-1 or anti-PD-L1, and 5 anti-CTLA-4 monotherapy. All patients initially received corticosteroids (1-2mg/kg/day methylprednisone equivalent). Thirteen patients (36%) were steroid relapsers. Consistent steroid response was seen in 18 (50%). Age, sex, liver metastases, prior ICI exposure, peak ALT or starting dose of steroids (≤1.5 vs >1.5mg/kg/day methylprednisolone equivalents) did not predict relapse, although relapsers were more likely to have been treated with combination anti-CTLA-4/PD-1 (7 (54%) relapsers, vs 3 (16%) responders, p = 0.02). Relapse occurred after a median of 14.5 days (range 8-111), and after taper to median 54% (5-100) of initial steroid dose. In responders, ALT normalisation occurred after median 14 days (range 3-56). In 27 patients where sufficient data were available, societal guidelines on ALT thresholds to initiate steroid taper were followed in 13 (48%; 6 relapsers and 7 responders). However, initiation of steroid taper was delayed in responders compared to relapsers (after median 7 days (2-15) in responders vs 4 days (range 2-9) in relapsers, p = 0.04). Overall, 5 relapsers responded to re-escalation of steroids. Eight required additional treatment with MMF, and 4 required 3^rd^ line therapy with Tacrolimus. Ultimately, hepatitis resolved in all patients.

**Conclusion(s):**

In patients with ICI hepatotoxicity, combination ICI therapy confers a higher risk of steroid relapse than monotherapy. There is significant heterogeneity in management of steroid dosing in patients with ICI hepatotoxicity. Delayed initiation of steroid taper may be associated with a reduced risk of relapse and warrants prospective evaluation as part of a standardised management algorithm.

**Please acknowledge all funding agencies by checking the applicable boxes below:**

None

**Disclosure of Interest:**

None Declared

